# Study protocol for OtoSurg 1: A prospective evaluation of worldwide tonsillectomy indications, techniques, and outcomes

**DOI:** 10.1371/journal.pone.0349700

**Published:** 2026-06-01

**Authors:** Zachary Elwell, Emma Finnegan, Kartik Motwani, Juyun Hwang, Caretia Washington, Katharine G. Johnson, Audrey Y. Su, Rolvix H. Patterson, Zhen Jason Qian, Catherine A. Shaw, Taseer Din, Maxwell P. Kligerman, on behalf of the Global Otolaryngology-Head and Neck Surgery Initiative

**Affiliations:** 1 Department of Otolaryngology-Head and Neck Surgery, University of Arizona College of Medicine – Tucson, Tucson, Arizona, United States of America; 2 Department of Otolaryngology-Head and Neck Surgery, Trinity College Dublin, Dublin, Ireland; 3 Department of Otolaryngology-Head and Neck Surgery, University of Florida College of Medicine, Gainesville, Florida, United States of America; 4 Renaissance School of Medicine, Stony Brook University, Stony Brook, New York, United States of America; 5 Department of Epidemiology, University of Florida, College of Public Health and Health Professions, Gainesville, Florida, United States of America; 6 University of Arizona College of Medicine – Tucson, Tucson, Arizona, United States of America; 7 Department of Biostatistics, Brown University, Providence, Rhode Island, United States of America; 8 Department of Head and Neck Surgery and Communication Sciences, Duke University School of Medicine, Durham, North Carolina, United States of America; 9 Department of Otolaryngology-Head and Neck Surgery, UC San Diego School of Medicine, San Diego, California, United States of America; 10 Centre for Surgical Informatics, University of Edinburgh, Edinburgh, United Kingdom; 11 Division of Pediatric Otolaryngology-Head and Neck Surgery, Sidra Medicine, Al Rayyan, Doha, Qatar; 12 Department of Otolaryngology-Head and Neck Surgery, Weill Cornell Medicine, Al Rayyan, Doha, Qatar; 13 Department of Otolaryngology-Head and Neck Surgery, Emory University, Atlanta, GeorgiaUnited States of America; Universiti Malaya Fakulti Perubatan: University of Malaya Faculty of Medicine, MALAYSIA

## Abstract

**Background:**

Tonsillectomy is the most common pediatric surgery performed globally, yet little is known about how tonsillectomy outcomes vary worldwide.

**Preliminary Data:**

Although generally considered a safe surgery, tonsillectomy carries significant risks. Approximately 3% of US children require hospital readmission within 30 days due to life-threatening complications. While mortality rates in high-income countries (HICs) are relatively low (~0.0005%), mortality rates in low- and middle-income countries (LMICs) are not well characterized, with some reports suggesting rates many times higher (~3%).

**Research gap:**

Despite the frequency and significance of tonsillectomy, it remains unclear how surgical indications, techniques, and settings may impact outcomes worldwide. In addition, there is currently no mechanism to perform a prospective investigation of global tonsillectomy outcomes.

**Hypotheses and specific aims:**

We hypothesize that the morbidity and mortality of routine tonsil surgery vary significantly according to geographic region and healthcare setting, as well as surgical indications and techniques. We aim 1) to quantify global variation in 30-day major postoperative complications and mortality following pediatric tonsillectomy across World Health Organization (WHO) regions and 2) to characterize global variation in pediatric tonsillectomy surgical indications and operative techniques across WHO regions.

**Design:**

OtoSurg 1 will be an international, multi-site, prospective cohort study. The central IRB is approved and hosted by Emory University (IRB ID: STUDY00009113). All healthcare facilities globally that perform pediatric tonsillectomy will be invited to participate. Participating investigators will be trained to collect data on patients undergoing primary tonsillectomy over a 90-day period at their respective institutions. Site-based teams will record demographic data, surgical indication(s), surgical technique(s), postoperative complications, and mortality directly into a global de-identified tonsillectomy outcomes data registry. We will leverage existing relationships and professional networks to recruit participants from 30 sites in each of the six WHO regions for a target of 180 partner sites.

**Potential impact:**

This study will highlight potential opportunities for intervention to standardize and improve outcomes for the world’s most common pediatric surgery, focusing on LMICs. Beyond its primary objectives, this project will also seek 1) to recruit and train a collaborative research network of otolaryngology surgeons from around the world and 2) to build the digital infrastructure necessary to support this network sustainably. This collaborative research network will serve as the foundation for future large-scale research initiatives and for the development and implementation of novel, data-driven interventions.

## Introduction

The Global Otolaryngology-Head and Neck Surgery (OHNS) Initiative is a 501(c)(3) nonprofit organization that aims to perform global, collaborative public health research with the aim of better understanding and expanding access to otolaryngology care worldwide [1]. The Global OHNS Initiative has published expert consensus-based studies that identified priority global OHNS conditions and procedures in both adult and pediatric populations [2,3]. Examples of the procedures identified as global priorities were tonsillectomy, thyroidectomy, and tracheostomy. The significant and disparate global burden of otolaryngology conditions underscores the need to conduct robust international studies to identify opportunities for intervention and improve health outcomes [4–6].

Conducting meaningful global outcomes research in otolaryngology requires coordinated, large-scale collaboration across diverse healthcare settings. The GlobalSurg Collaborative (GlobalSurg) serves as a model for the design of this study. GlobalSurg, the product of the UK National Institute of Health Care Research’s Global Health Research Unit on Global Surgery, is a robust international community that produces high-impact, prospective, multi-site research studies to advance surgical care [7–9]. In 2016, GlobalSurg published a landmark article assessing worldwide emergency abdominal surgery outcomes, establishing a precedent for the design of collaborative global international surgical outcomes research [7]. This study builds on the successful GlobalSurg model and will help quantify potential disparities in global pediatric tonsillectomy outcomes.

### Study topic selection

Candidate procedures for OtoSurg 1 were first identified by the study authors using the Global OHNS Initiative’s consensus priority procedures, which are widely performed across global settings, delivered by OHNS providers, and easily measurable [2]. Candidate procedures were discussed at two separate open forum Global OHNS Initiative meetings, and there was a collective agreement supporting the selection of pediatric tonsillectomy. Although tonsillectomy is the most common pediatric surgery worldwide, there is limited understanding of how its indications, techniques, and outcomes vary across diverse healthcare settings [10]. Findings from the Global OHNS Initiative studies highlight its importance: tonsillectomy and control of post-tonsillectomy hemorrhage were ranked among the top consensus procedures for pediatric otolaryngology [3]. At the same time, tonsillar hypertrophy and obstructive sleep apnea were identified as the 3^rd^ and 6^th^ most critical pediatric conditions, respectively [3].

Although generally considered a safe surgery, tonsillectomy carries significant risks for children. Approximately 3% of children in the United States (US) require hospital readmission within 30 days due to life-threatening complications [11,12]. While mortality rates in high-income countries (HICs) are low (~0.0005%), mortality rates in low- and middle-income countries (LMICs) are not well characterized, with some reports suggesting rates that are orders of magnitude higher (~3%) [13–15]. New techniques such as intracapsular tonsillectomy and radiofrequency plasma ablation (e.g., Coblator or COBLATION Technology (Trademark of Smith & Nephew PLC, Watford, England)) tonsillectomy may reduce the incidence of complications [16–20]. However, it is unclear how these techniques are utilized across different settings and whether they are associated with specific postoperative outcomes.

The indications, techniques, and postoperative outcomes for pediatric tonsillectomy are well documented in the US, as evidenced by the updated clinical practice guideline from the American Academy of Otolaryngology – Head and Neck Surgery, published in 2019 [12]. A 2015 systematic review and meta-analysis of adenotonsillectomy complications identified respiratory compromise and secondary hemorrhage as the most common early complications globally [13]. However, country-specific clinical practice guidelines and investigations of postoperative complications remain limited in many settings. Several reports in the literature provide insight into current practices in LMICs. For example, Muninnobpamasa et al. described the prevalence of post-tonsillectomy complications at a single institution in Thailand, Kligerman et al. found that tonsillectomy (with or without adenoidectomy) was the most frequently performed OHNS surgery at a single institution in Haiti, and Onotai et al. reported that preoperative blood grouping and cross-matching before adenoid and tonsil surgeries were often not cost-effective or relevant in most circumstances at a single institution in Nigeria [14,21,22]. Additional reports from HICs highlight knowledge gaps. For example, Murto et al. identified that rates of adverse post-adenotonsillectomy outcomes and associated factors were poorly described in Canada, indicating a need for standardized perioperative treatment algorithms [15].

Building on this background, OtoSurg 1 will be an international, multi-site, prospective cohort study that will describe global variations in indications, techniques, and outcomes for pediatric tonsillectomy. Additionally, this study will provide a framework for future multinational, prospective collaborations to be replicated across the global OHNS research community. Ultimately, this research network is intended to improve access to high-quality otolaryngology care globally.

## Materials and methods

### Aims

The primary aims of this project are 1) to quantify global variation in 30-day major postoperative complications and mortality following pediatric tonsillectomy across World Health Organization (WHO) regions and 2) to characterize global variation in pediatric tonsillectomy surgical indications and operative techniques across WHO regions.

### Hypothesis

We hypothesize that the morbidity and mortality associated with routine tonsillectomy, as well as surgical indications and techniques, vary significantly across different geographic regions and healthcare facilities.

### Study design, setting, and site eligibility

**Fig 1:**
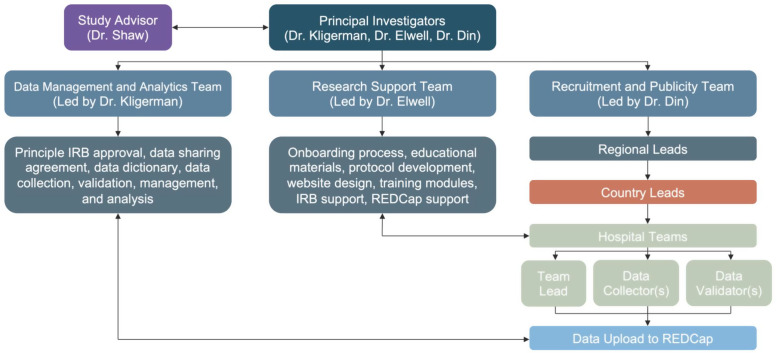
OtoSurg 1 organizational structure. The OtoSurg Leadership Team will consist of three groups: Recruitment and Publicity, Research Support, and Data Management and Analytics. The Recruitment and Publicity Team will oversee all WHO regional leads, individual country leads, and specific hospital teams. The Research Support Team will manage the onboarding process for new OtoSurg members, develop educational materials, and provide IRB and REDCap assistance. The Data Management and Analytics Team will collect, validate, and analyze all study data. By working closely together, these three teams will serve as the organizational framework of OtoSurg 1.

OtoSurg 1 will be an international, multi-site, prospective cohort study. This study will follow a methodology similar to that previously developed and implemented by GlobalSurg [23,24]. The OtoSurg Leadership Team will consist of three groups: Recruitment and Publicity, Research Support, and Data Management and Analytics (Fig 1). The Recruitment and Publicity Team will oversee all WHO regional leads, individual country leads, and specific hospital teams. The Research Support Team will manage the onboarding process for new OtoSurg members, develop educational materials, and assist with IRB and REDCap use. The Data Management and Analytics Team will collect, validate, and analyze all study data. By working closely together, these three core teams will serve as the organizational framework of OtoSurg 1.

**Fig 2:**
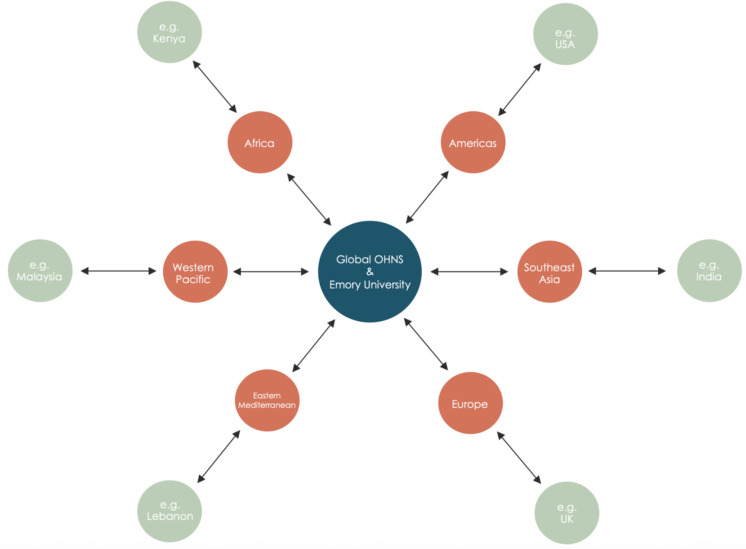
OtoSurg 1 hub-and-spoke model. The Global OHNS Initiative and Emory University will serve as the central hub (blue), and regional representatives in the six WHO regions (orange) will act as liaisons to individual partner sites (green).

The broader OtoSurg network will be organized utilizing a hub-and-spoke model, with the Global OHNS Initiative and Emory University serving as the central hub, linking to regional representatives across the six WHO regions and individual researchers at country-specific partner sites (Fig 2). Any healthcare facilities worldwide where pediatric tonsillectomies are performed will be eligible to participate (except sites in the People’s Republic of China (PRC) due to the Personal Information Protection Law (PIPL) of the PRC). The study will aim for balanced representation from each of the six WHO regions.

Each participating research site must have between two and four investigators, fulfilling the required roles of Data Recorder, Data Validator, and Team Lead. The Data Recorder and Data Validator must be two separate individuals. However, either of these individuals may also serve as the Team Lead. The Team Lead will act as the primary point of contact with the central coordinating hub. Whenever possible, each team is encouraged to include at least one attending physician from the participating site. The attending physician may serve as the Data Recorder, Data Validator, or Team Lead, or may instead participate in a more general advisory capacity to support the team. Participating sites must obtain IRB/ethics committee approval or exemption and have access to the shared REDCap database. Aside from these requirements, there are no strict inclusion or exclusion criteria for sites. There is no minimum number of patients required to be recorded per facility or healthcare setting.

A team will capture data on all primary pediatric tonsillectomy cases performed at their site during the designated 90-day study period – even if not every case is performed by the specific attending physician listed on the project. The attending physician listed on the project will serve as the representative for the entire division or site. For example, at Emory University, the Division of Pediatric Otolaryngology includes seven faculty members. Even if only one faculty member is designated as the attending physician on the Emory OtoSurg 1 team during a 90-day data collection period, we encourage the inclusion of all tonsillectomy cases performed by any member of the division during that time frame. This approach will help ensure comprehensive data capture and avoid selection bias. However, the final decision regarding which cases to include will be at the discretion of each participating site and is subject to their local IRB/ethics committee approval process. Every member of each research team will be listed as an author on the final manuscript, except those who do not meet the data validation criteria outlined below.

### Timeline

**Fig 3:**
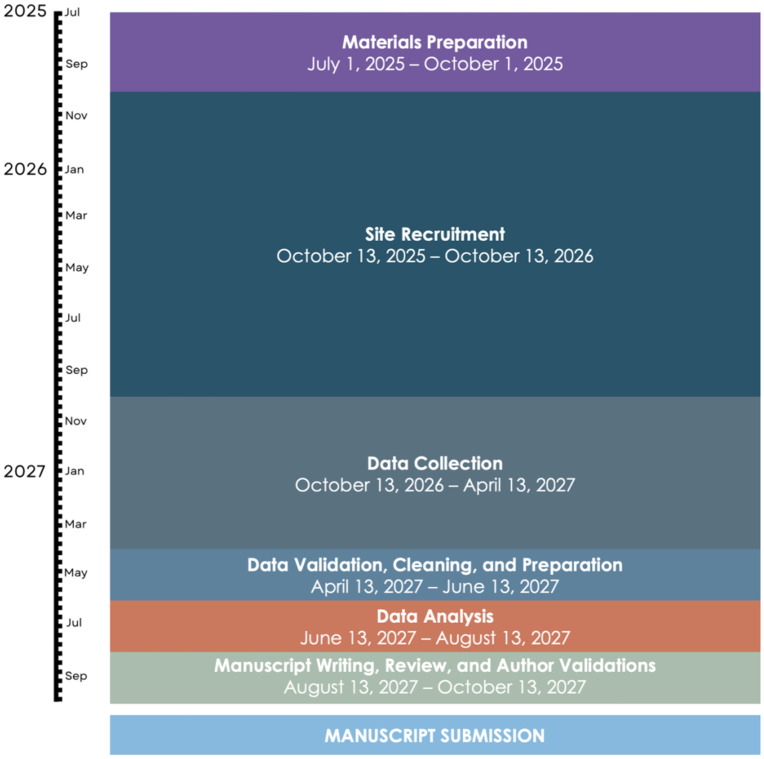
OtoSurg 1 timeline. Overview of the OtoSurg 1 project timeline.

The study will begin with recruiting and training participating sites. Recruitment, training, and individual site IRB/ethics committee approval will occur over 12 months from October 13, 2025, to October 13, 2026 (Fig 3). The six-month data collection period will be from October 13, 2026, to April 13, 2027. Individual sites will be able to select any 90-day interval during this data collection window to record and submit their data. Data validation, cleaning, and preparation will occur over two months from April 13, 2027, to June 13, 2027. Data analysis will occur over two months, from June 13, 2027, to August 13, 2027. Results are expected to be complete by August 13, 2027. Manuscript writing, review, and author validations will occur over two months, from August 13, 2027, to October 13, 2027. We anticipate submitting the final manuscript in late 2027 or early 2028.

### Study population and inclusion/exclusion criteria

The study population will comprise all consecutive patients under 18 years old undergoing primary tonsillectomy during the specified study period at the participating site. Tonsillectomy will be defined as the removal or ablation of any amount of palatine tonsil tissue using any surgical method. Both unilateral and bilateral tonsillectomy cases are eligible. Patients undergoing concurrent procedures, such as adenoidectomy or tympanostomy tube placement, will be included. Sites from any country worldwide are eligible to participate, excluding the PRC due to the PIPL.

Country classification by income level will be defined using the World Bank income stratification which is derived from gross national income (GNI) per capita and the World Bank Atlas method [25,26]. Using this method, low-income countries (LICs) are defined as those with a GNI per capita ≤1,135 (USD), lower middle-income countries (LMICs) are those with a GNI per capita ≥1,136 and ≤4,495 (USD), upper middle-income countries (UMICs) are those with a GNI per capita ≥4,496 and ≤13,935 (USD), and high-income countries (HICs) are those with a GNI per capita >13,935 (USD) [25,26].

### Outcome measures

The primary outcome measures will include the distribution of pediatric tonsillectomy surgical indications and techniques across WHO regions and the rate of major complications and postoperative mortality within 30 days of tonsillectomy. Major complications will be defined as hospital readmission, need for unplanned surgical intervention, or postoperative hemorrhage. Hospital readmission will be defined as any admission to the hospital within 30 days of the date of surgery and after the initial surgical admission for tonsillectomy or presentation on the day of surgery for children undergoing tonsillectomy as an outpatient procedure. Need for unplanned surgical intervention will be defined as any return to the operating room for surgical intervention within 30 days of the date of surgery. Postoperative hemorrhage will be defined as any amount of postoperative bleeding that prompts the patient to return to the hospital, seek additional medical care, require further medical intervention, or delay hospital discharge. Postoperative mortality will be defined as any death within 30 days of the date of surgery, initial surgical admission, or during a prolonged initial surgical admission lasting more than 30 days [27]. Partner sites will be responsible for reporting mortality in accordance with their local institutional policies.

### Data abstraction and recording

Each participating site will prospectively collect and record data over a consecutive 90-day period of its choosing within the overall 6-month data collection window. Within the 90-day data collection period, new patient accrual will occur over the first 60 days. Only postoperative information on previously enrolled patients will be recorded during the last 30 days. Participating teams will implement site-specific strategies to ensure the identification and inclusion of all eligible patients. These strategies may include daily review of operating room logbooks, multidisciplinary team meetings, and review of surgical admission or handover lists. Patient-level data will be obtained through a review of medical records, discussions with clinical team members, or, where appropriate, communication with patients and their families during routine clinical care. Data on postoperative complications will be collected based on return visits to the clinic, emergency room, and/or hospital within 30 days following surgery. Teams will not be required to contact patients and families individually to inquire about postoperative complications. The following patient data will be collected: age, sex, anesthesia preoperative risk class, tonsillectomy indication, tonsillectomy technique (intracapsular vs. extracapsular; Bovie/monopolar diathermy vs. bipolar diathermy vs. radiofrequency plasma ablation vs. microdebrider vs. cold steel vs. other), 30-day postoperative major complications, and 30-day postoperative mortality. In addition, data on participating sites will be collected, including WHO region, country, WHO health care facility level, and country World Bank income group [28]. The data variables and specific survey questions were developed using iterative feedback from representatives of each of the six WHO regions. Further information pertaining to anesthesia preoperative risk class, indications for surgery, and surgical instrumentation may be found in Appendix A. A complete list of captured data elements and parameters may be found in Appendix B.

The OtoSurg team will offer two options for data recording: either a hard copy or an electronic form. Sites can use either method or a combination of both, depending on their local workflows. All data must be recorded in a de-identified format using a unique study identifier (ID). Each site is responsible for maintaining a secure local linkage file that connects study IDs to individual patient identifiers. This file will remain confidential and will not be shared with the OtoSurg team.

### Data submission

Data submission will be completed using a secure, password-protected REDCap survey link provided by the OtoSurg team. This link will only be accessible to sites that have received IRB approval or exemption to participate. Data for each patient must be submitted no earlier than 30 days after their surgery date to ensure complete 30-day outcome reporting. Sites can choose to submit data continuously, in batches, or all at once, but all data must be submitted within 90 days of the site’s initial data collection start date. Once a REDCap survey response is submitted, it cannot be edited or revised, so sites must ensure completeness and accuracy before submission.

### Data validation

Each participating site will designate one team member as the Data Validator. This person will independently record the total number of tonsillectomy cases that meet the inclusion criteria during the specified data collection period at their site. This total will be referred to as the total case ascertainment. Data Validators can gather this information either prospectively or retrospectively, depending on what is most practical for their site’s workflow and record-keeping systems. Sites missing data for more than 20% of patients included during their 90-day collection period will be excluded from the final analysis and will be ineligible for authorship.

The Data Validator will be randomly assigned five cases from their team to review. The Data Management and Analytics team will randomly assign these cases and provide the Data Validator with the submitted data. If fewer than five cases are available, all eligible cases will be reviewed. To perform the review, the Data Validator will obtain the de-identified study data and the local linkage file from the Data Recorder, which will be in the form of a Microsoft Excel file or a hard copy paper file. Using this information, the Data Validator will conduct a retrospective chart review for these cases to evaluate the accuracy and completeness of the recorded data. The Data Validator will submit a separate, password-protected REDCap survey link provided by the OtoSurg team. This survey will ask the Data Validator to assess the accuracy and completeness of the recorded data and determine if patients had postoperative follow-up.

Sites must demonstrate ≥80% 30-day follow-up completeness among enrolled patients to remain eligible for analysis. Essential variables required for inclusion in the primary analysis include age, sex, anesthesia risk class, surgical indication, surgical technique (i.e., intracapsular vs. extracapsular), device type (i.e., Bovie/monopolar diathermy vs. bipolar diathermy vs. radiofrequency plasma ablation vs. microdebrider vs. cold steel vs. other), and 30-day complication status. Cases missing primary outcome data will be excluded from outcome analysis but retained for descriptive reporting. Sensitivity analyses will be performed excluding sites with <90% follow-up completeness.

### Recruitment and training

An open invitation to participate in OtoSurg 1 will be broadly shared through personal contacts, professional email listservs, and existing national and international networks. Regional and country leads will be recruited who can provide a detailed site-specific understanding to support recruitment and training. The Recruitment and Publicity Team will partner directly with the Global OHNS Initiative’s extensive network of LMIC and HIC providers in addition to collaborating with otolaryngology professional society leaders worldwide to promote balanced representation from all WHO regions.

The OtoSurg team will provide research training and access to a shared REDCap database. They will also assist with participant recruitment and IRB processes. Training resources will include live webinars, PDF documents (including a dedicated IRB support guide), and pre-recorded videos developed for this study. Training materials will be offered in English, with translations into partner site languages as resources permit. Training materials will be hosted on a central website to facilitate access by participants globally (https://www.globalohns.org/research-team-resources/otosurg).

### Power analysis and statistical analysis plan

Aim 1 (to quantify global variation in 30-day major postoperative complications and mortality following pediatric tonsillectomy across WHO regions) is inferential and will evaluate associations between country income (HICs and LMICs) and 30-day outcomes, including major complications and mortality.

Aim 2 (to characterize global variation in pediatric tonsillectomy surgical indications and operative techniques across WHO regions) will be purely descriptive and does not require power analysis.

We aim to recruit 30 individual sites from each of the six WHO regions (a target of 180 total sites) to ensure the data is globally diverse and representative of each WHO region. The rate of postoperative complications is estimated to be approximately 5% greater in LMICs than in HICs [13,16,23,24]. We anticipate an unbalanced sampling ratio of 70% HIC and 30% LMIC participants. Assuming a two-sided α = 0.05, 80% power, and an anticipated enrollment ratio of 70% HIC and 30% LMIC participants, the required independent-sample size is approximately 5,770 patients. To account for clustering of patients within hospitals, assuming an average of 20 patients per site during the 60-day accrual period and a modest degree of within-hospital correlation, we inflated the target sample size accordingly. After additionally accounting for an anticipated 5% rate of mixing 30-day outcome data, the final target sample size is approximately 7,200–7,500 patients, corresponding to roughly 360–375 participating hospitals. Regarding the secondary outcome of mortality, our literature review suggests a maximum mortality rate of 3% in LMICs, compared to a maximum rate of 0.01% in HICs [10,21]. Assuming the same parameters as above, we must recruit data from at least 4,260 participants to detect a difference between HIC and LMIC populations.

We will use multilevel mixed-effects logistic regression to account for clustering of patients within hospitals and hospitals within countries. For each primary outcome (30-day major complication and 30-day mortality), we will fit a sequence of prespecified models. First, we will fit an unconditional (null) model with random intercepts for hospital and, where there are sufficient numbers of hospitals per country to permit stable variance estimation, for country to quantify baseline between-hospital and between-country variation. Second, we will add prespecified patient- and procedure-level covariates based on clinical relevance and prior literature (i.e., age, sex, anesthesia preoperative risk class, surgical indication, surgical technique, device type, and major medical comorbidities) to evaluate the extent to which case-mix explains outcome variation. Third, we will include country income classification (HIC vs. LMIC) as a fixed effect to estimate the adjusted association between income setting and outcomes while retaining the hierarchical random-effects structure. Model fit will be assessed using Akaike information criterion (AIC). Intraclass correlation coefficients (ICCs) will be calculated to quantify the proportion of outcome variance attributable to clustering at the hospital (and country) level for dichotomous outcomes using the logistic distribution variance. Adjusted odds ratios with 95% confidence intervals will be reported.

### Ethical concerns, governance, and consent process

There are no specific ethical concerns regarding this project. All data will be observational, de-identified, and will not impact patient clinical care. Individual IRB/ethics committee approval or exemption will be required for each participating site. All collaborators will be appropriately trained and supported with guidance on how to seek local IRB/ethics committee approval or exemption. All members of research teams at participating sites will receive authorship credit on any manuscripts produced in accordance with the previously defined authorship criteria and the Global OHNS Initiative Research Equity Guidelines [29].

OtoSurg 1 will follow a centralized coordination with decentralized regulatory oversight model, consistent with prior global collaborative studies [7,8,30–33]. Emory University serves as the central coordinating hub and REDCap data repository, responsible for overall study design, data infrastructure, and data management. However, all regulatory and ethical responsibilities – including IRB/ethics committee approval, consent procedures, and mortality reporting – are governed at the level of each participating site in accordance with local regulations and institutional policies.

The authorship team believes a waiver of informed consent is valid for this study, and a waiver has been granted by the central hub IRB/ethics committee at Emory University. Informed consent was waived on the basis that the proposed data collection involves no increased risk to study subjects. Like most chart review studies, this project involves a review of medical records, which, by nature, contain protected health information; however, no direct interventions, treatments, or interactions with participants will occur. The medical record will be reviewed, and de-identified data will be recorded directly into REDCap, ensuring that there is no potential for physical, psychological, or social harm to participants. However, each individual participating site needs to make its own determination in accordance with local IRB/ethics committee requirements.

In anticipation of partner sites whose IRB/ethics committees may deny a waiver of informed consent and require written consent, we have provided a standardized, plain-language patient information sheet and written participant consent form, both of which are available on the OtoSurg website (https://www.globalohns.org/research-team-resources/otosurg). All study participants will be children <18 years old. As such, for partner sites that require written consent, consent will be obtained according to the consent procedures of the participating site in accordance with local regulations and institutional policies.

## Discussion

Our study aims to quantify global variation in 30-day major postoperative complications and mortality following pediatric tonsillectomy cases globally, identifying gaps and highlighting targetable areas for improving outcomes across varying settings. Additionally, the secondary aims of this study are 1) to recruit and train a collaborative research network of otolaryngology surgeons and trainees from around the world who will be poised to carry out future research projects and 2) to build the digital infrastructure necessary to support this work sustainably.

### Potential difficulties and limitations

Similar methodological studies have been successfully conducted in the general surgery literature [7–9,30–45]. A potential challenge will be recruitment and participation in LMICs. We have accounted for this by utilizing an unbalanced sampling ratio of 70% HICs to 30% LMICs and allocating 12 months for site recruitment. Additionally, we have selected the Global OHNS Initiative as a central hub to facilitate outreach to and relationships with LMIC partners.

Another challenge will be ensuring data accuracy. For this reason, we are devoting significant time and effort to training collaborators. We have developed research training that follows well-established principles, encouraging participating sites to implement data extraction methods that are contextually appropriate for their institutions [9]. Additionally, we will require each site to have a designated Data Validator who will audit results to ensure data completion and accuracy.

A small portion of complications and/or mortalities may be missed if patients do not return to the same hospital where they had their initial surgery. This is an inherent limitation of the study. When reviewing cases, the Data Validator will assess the number of postoperative patients who had direct follow-up, regardless of their complication status. Additionally, velopharyngeal insufficiency and velopharyngeal and/or oropharyngeal stenosis are important major complications that are more likely to occur with poor surgical technique, and which may be underreported in some regions. However, these complications typically take more than 30 days to become clinically evident in the postoperative period. As such, patients who develop these complications may be missed.

Surgeon experience is an important determinant of postoperative outcomes. However, in many participating centers, especially teaching hospitals, patient care is delivered by diverse teams involving attending surgeons, trainees, and other providers, making it difficult to attribute outcomes to a single surgeon or quantify operative experience in a standardized fashion across sites. For these reasons, surgeon-level variables will not be captured, an inherent limitation of the study.

## Supporting information

S1 File Appendix A: Perioperative classifications, indications, and instruments. (PDF)

S2 File Appendix B: Data dictionary. (DOCX)
