# Supplementary material for: Study protocol for OtoSurg 1: A prospective evaluation of worldwide tonsillectomy indications, techniques, and outcomes
Source: PLoS One. 2026 Jun 1;21(6):e0349700. doi: 10.1371/journal.pone.0349700 (PMC13225423; doi:10.1371/journal.pone.0349700)
Supplement: S1 File — (PDF) [file pone.0349700.s001.pdf]

Appendix A: Perioperative classifications, indications, and instruments

American Society of Anesthesiologists (ASA) classification: [45]

ASA Classification	Definition	Example
I	A normal healthy patient.	Healthy, non-smoking, no or minimal alcohol use.
II	A patient with mild systemic disease.	Mild diseases only without substantive functional limitations.<br>Current smoker, social alcohol drinker, pregnancy, obesity<br>(30<BMI<40), well-controlled DM/HTN, mild lung disease
III	A patient with severe systemic disease.	Substantive functional limitations; One or more moderate to<br>severe diseases. Poorly controlled DM or HTN, COPD, morbid<br>obesity (BMI ≥40), active hepatitis, alcohol dependence or<br>abuse, implanted pacemaker, moderate reduction of ejection<br>fraction, ESRD undergoing regularly scheduled dialysis,<br>history (>3 months) of MI, CVA, TIA, or CAD/stents.

IV	A patient with severe systemic disease that is a constant threat to life.	Recent (<3 months) MI, CVA, TIA or CAD/stents, ongoing cardiac ischemia or severe valve dysfunction, severe reduction of ejection fraction, shock, sepsis, DIC, ARD or ESRD not undergoing regularly scheduled dialysis.
V	A moribund patient who is not expected to survive without the operation.	Ruptured abdominal/thoracic aneurysm, massive trauma, intracranial bleed with mass effect, ischemic bowel in the face of significant cardiac pathology or multiple organ/system dysfunction.

Abbreviations:

DM Diabetes Mellitus

HTN Hypertension

COPD Chronic Obstructive Pulmonary Disease

ESRD End-Stage Renal Disease

MI Myocardial Infarction

CVA Cardiovascular Accident

TIA Transient Ischemic Attack

CAD Coronary Artery Disease

DIC Disseminated Intravascular Coagulation

ARD Acute Respiratory Distress

Indications for surgery: [12]

Indications	Definition
Recurrent throat infections	≥7 episodes in past year, ≥5 episodes per year for 2 years, or ≥3 episodes per year for 3 years; each episode should have clinical features (fever, cervical adenopathy, tonsillar exudate, or positive group A strep test). May also consider surgery with modifying factors (severe episodes, antibiotic allergies, significant school absences).
Obstructive sleep-disordered breathing (oSDB)	Clinical diagnosis: obstructive abnormalities of respiratory pattern or oxygenation/ventilation during sleep (snoring, mouth breathing, apneas). Includes spectrum from primary snoring to obstructive sleep apnea (OSA). Associated symptoms: inattention, poor concentration, hyperactivity, excessive sleepiness, growth failure, enuresis, behavioral problems.

Tonsil grading: [12,46]

**Brodsky Grading**	**Definitions**
Grade I (Tonsils hidden within pillars)	Tonsils occupy <25% of airway
Grade II (Tonsils extend to pillars)	Tonsils occupy 25–50% of airway
Grade III (Tonsils extend beyond pillars)	Tonsils occupy 50–75% of airway
Grade IV (Tonsils extend to midline)	Tonsils occupy >75% of airway

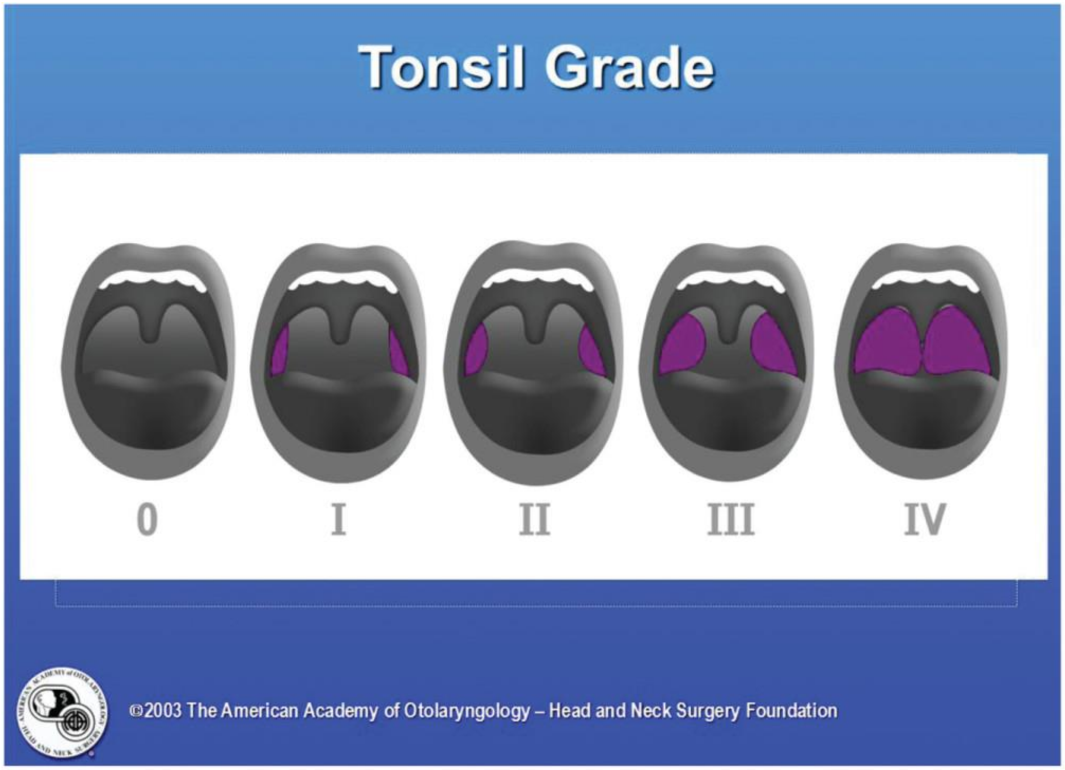

Complications: [12]

**Complications**	**Definitions**
Primary bleeding	Bleeding occurring within 24 hours of surgery
Secondary bleeding	Bleeding occurring more than 24 hours after surgery
Respiratory compromise	Airway obstruction, laryngospasm, or hypoxemia requiring intervention
Pain	Significant throat pain postoperatively, often requiring analgesia
Dehydration	Inadequate oral intake leading to clinical dehydration, sometimes requiring IV fluids
Nausea and vomiting	Postoperative emesis, may delay oral intake and discharge
Trauma to adjacent structures	Injury to teeth, lips, tongue, pharyngeal wall, or soft palate during surgery
Velopharyngeal insufficiency	Hypernasal speech or nasal regurgitation due to palatal dysfunction
Post obstructive pulmonary edema	Pulmonary edema following relief of chronic upper airway obstruction
Referred otalgia	Ear pain due to shared nerve pathways with the oropharynx
Death	Rare, but may result from severe bleeding or airway compromise

52 # Instruments:

Device	Image
Radiofrequency Plasma Ablation	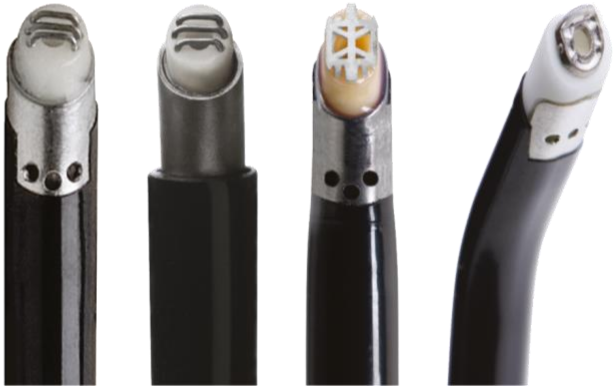
Snare	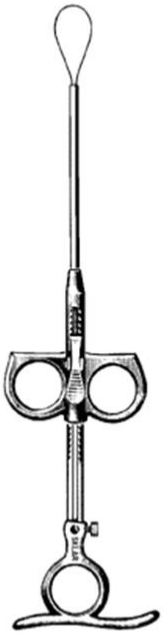

Curette	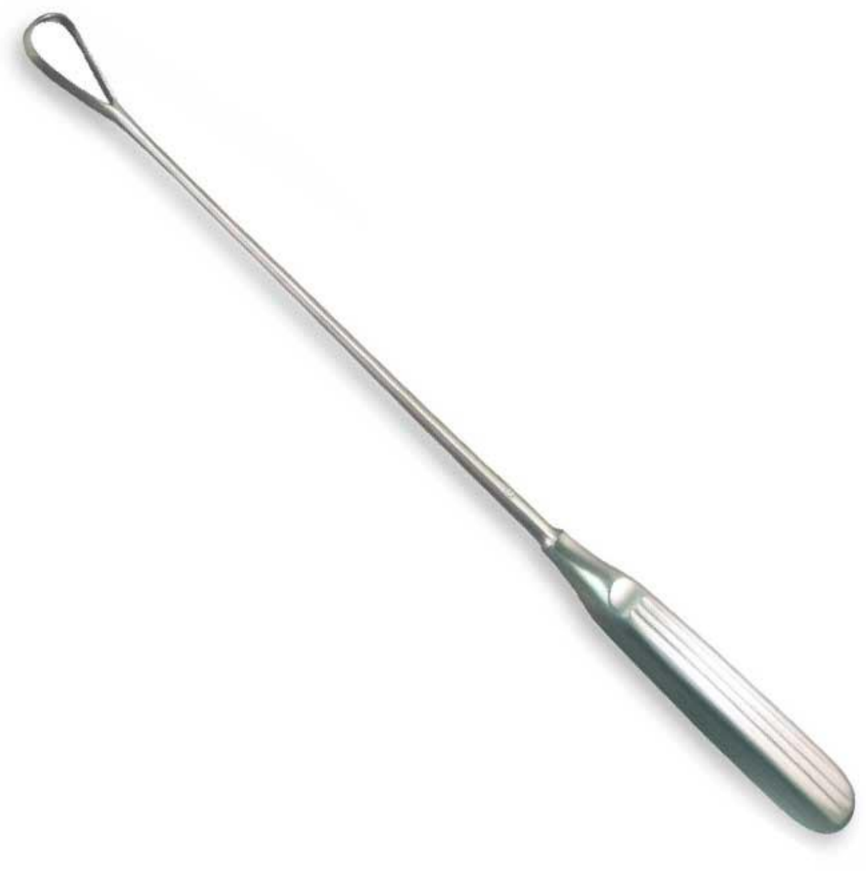
Microdebrider	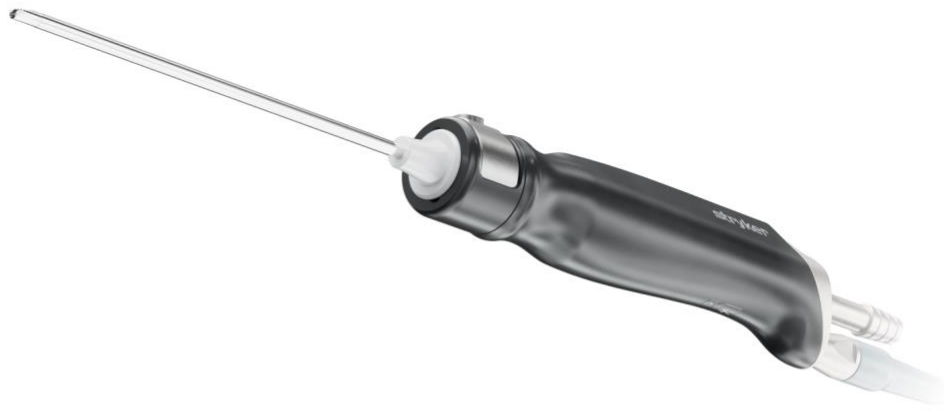

Bovie/mono-polar cautery	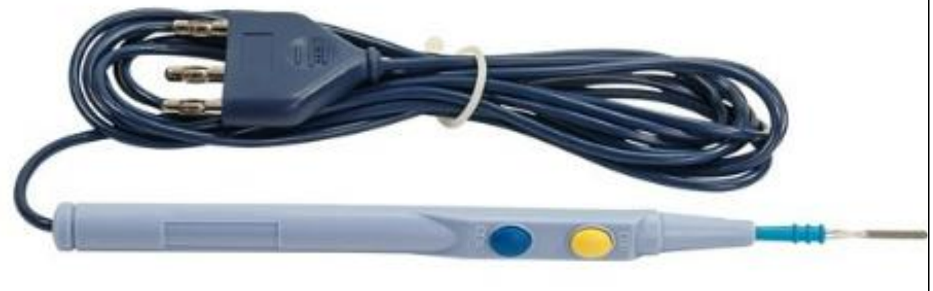
Suction Bovie	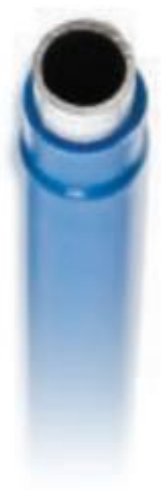
Scalpel	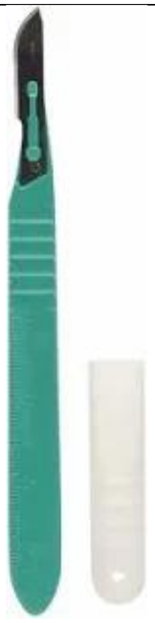

53
