# Supplementary material for: Study protocol for OtoSurg 1: A prospective evaluation of worldwide tonsillectomy indications, techniques, and outcomes
Source: PLoS One. 2026 Jun 1;21(6):e0349700. doi: 10.1371/journal.pone.0349700 (PMC13225423; doi:10.1371/journal.pone.0349700)
Supplement: S2 File — (DOCX) [file pone.0349700.s002.docx]

**Appendix B: Data dictionary**

| **Pediatric Tonsillectomy Case** | **Required Data (description/comment)** |
| --- | --- |
| **Study Site Information** | |
| Site ID  (Unique ID for your study location.)  Note: Must star with 2 letters and end with 4 numbers. | Text, required |
| Email address for site study coordinator | Text (email), required |
| **Subject Demographics** | |
| Patient De-identified Study Number  (Unique deidentified code for the specific study subject) | Text, required |
| Sex at birth | Male / Female / Non-binary / Not reported |
| Subject Age  (age in years at time of procedure; please round to the nearest whole year) | Text (integer, Min: 0, Max: 120), Required |
| Body mass Index (BMI) (kg/m^2^) | (number; record to 1 decimal point) |
| **Subject Characteristics** | |
| Major Medical Comorbidities  (Health Conditions) | 1. Autoimmune condition (e.g., rheumatoid arthritis, systemic lupus erythematous, vasculitis) 2. Bleeding disorder 3. Cardiac disease (e.g. congenital heart defect or valvular disease) 4. Cerebral palsy 5. Chronic kidney disease 6. Craniofacial abnormalities (i.e. Cleft palate or cleft lip, Pierre Robin, craniosynostosis, hemifacial microsomia, etc.) 7. Developmental delay 8. Down Syndrome 9. Diabetes (Type 1 or 2) 10. Severe asthma/ hyperactive airway disease 11. Sickle cell disease 12. Other neurological disorder   99) Other  0) None  999) Data unavailable |
| If other major medical comorbidity was noted, please specify: | Text |
| Anesthesia preoperative risk class (ASA physical status classification)  (For additional information, please visit this link: [ASA physical status classification](https://www.asahq.org/standards-and-practice-parameters/statement-on-asa-physical-status-classification-system)):  ASA I: an otherwise healthy child.  ASA II: patient with mild systemic disease.  - For example, a child with mild asthma, mild obstructive sleep apnea or a well-managed abnormal heart rhythm.  ASA III: patient with severe systemic disease.  - For example, a child with severe asthma, a heart abnormality, epilepsy, or severe obstructive sleep apnea. ASA IV: patient with severe systemic disease that is a constant threat to life.  - For example, a child with heart failure or dependent on a ventilator. ASA V: moribund patient who is not expected to survive without surgery.  - For example, a child with a brain bleed or severe liver disease. | ASA I / ASA II / ASA III / ASA IV / ASA V |
| Tonsillectomy indication | 1. Recurrent acute tonsillitis / 2. Sleep disordered breathing and/or obstructive sleep apnea (either diagnosed clinically or via pre-operative testing) 3. Peritonsillar abscess (either acute or history of peritonsillar abscess) / 4. Halitosis or tonsilliths 5. Tonsillar hypertrophy interfering with eating, speaking, or breathing 6. Tonsillar asymmetry/ Concern for neoplasm 7. Other indication |
| If other indication, please specify. | Text |
| Were either a pre-operative overnight polysomnogram, pulse oximetry, or other similar testing performed? | No /  Yes- Polysomnogram/  Yes- Pulse oximetry/  Yes- Other preoperative overnight sleep testing/  Data unavailable |
| On pre-operative polysomnogram, what OSA severity was determined? | Mild (1-4) /Moderate (5-9) /  Severe (10 or greater) / Not applicable (NA)/ Data unavailable |
| From pre-operative overnight oximetry, please record value for oxygenic desaturation index (ODI): | Text |
| From pre-operative overnight oximetry, please record value for O2 nadir, if available: | Text |
| If other pre-operative sleep testing was performed, please record available test results: | Text |
| Please select grade of pre-operative palatine tonsillar hypertrophy: | Grade I (Tonsils hidden within pillars) /  Grade II (Tonsils extend to pillars) /  Grade III (Tonsils extend beyond pillars) /  Grade IV (Tonsils extend to midline)/  Data unavailable |
| **Operative procedure** | |
| Was an additional procedure (e.g., adenoidectomy, turbinate reduction, or other) performed at the time of tonsil surgery? | Yes/No |
| If yes, what additional procedure was performed? | Adenoidectomy/ Turbinate reduction/ Myringotomy with or without ear tube placement/ Other (open response) |
| If other procedure was performed at time of tonsillectomy, please indicate: | Text |
| Dissection Type | Intracapsular (partial/tonsillotomy) / Extracapsular (total) |
| What was the primary technique used for tonsillectomy/tonsillotomy?  (Please select the single answer that best describes the procedure) | Primary cold steel device (i.e. Snare or Scalpel or Microdebrider) /  Thermal powered device (i.e. Bovie/electrocautery or Radiofrequency plasma ablation) |
| If thermal powered device, what was the primary device used for procedure?  (Note: If multiple devices were used, select only the one that you feel represents the primary device.) | Bovie (unipolar)/ Suction Bovie / Radiofrequency plasma ablation / Bipolar / Other (open response) |
| If cold steel device, what was the primary device used?  (Note: If multiple devices were used, select only the one that you feel represents the primary device.) | Snare / Scalpel / Microdebrider / Fisher Tonsil Blade/ Hurd Dissector/ Other (open response) |
| What tools were used for hemostasis? (Please select all that apply) | Bovie (monopolar)/ Suction Bovie / Radiofrequency plasma ablation / Bipolar / Suture ligation/ Other (open response) |
| If other, please indicate what device was used for hemostasis: | Text |
| If other, please indicate what thermal powered device was used: | Text |
| If other, please indicate what cold steel device was used: | Text |
| What was the intraoperative estimated blood loss (EBL) for the procedure (in mL)? | Text (integer) |
| What was the patient's post-operative discharge status? | Same-day discharge to home/ Admitted to hospital overnight following surgery/ Other (please specify) |
| If patient was admitted postoperatively, please indicate level of care (floor, ICU) for admission: | Hospital floor bed/ Intensive care unit (ICU) |
| If postoperative discharge status is not listed above, please elaborate: | Text |
| Was an additional procedure (e.g., adenoidectomy, turbinate reduction, or other) performed at the time of tonsillectomy? | Yes / No |
| **Postoperative complications and 30-day course** | |
| 30-day postoperative major complication  (Select all that apply) | 1. Hospital re-admission / 2. Need for unplanned surgical intervention / 3. Post-operative hemorrhage (any amount of postoperative bleeding that prompts the patient to return to the hospital, seek additional medical care, require further medical intervention, or delay hospital discharge) / 4. Other |
| If you believe another major postoperative complication occurred that is not reflected in the above choices, please specify: | Text |
| If postoperative complication was hospital readmission, reason for re-admission:  (Please select all that apply) | 1. Poor oral intake (including  dehydration or vomiting) / 2. Pain control / 3. Tonsil bleeding / 4. Post-operative infection (e.g. lower respiratory tract infection)/ 5. Need for unplanned laryngo-tracheal intubation (distinct from intubation that may have been required for revision surgery) 6. Other |
| Management strategy for postoperative hemorrhage: | No intervention (observation) /  Conservative measures  (e.g. IV hydration, medication, or  direct pressure) /  Surgical control of bleeding  Other |
| Timing of postoperative hemorrhage | Primary (less than 24 hours from completion of surgery)/  Secondary (greater than 24 hours from completion of surgery) |
| If other intervention for postoperative hemorrhage, please specify: | Text |
| Postoperative 30-day mortality/ death | Yes / No |
| Please describe postoperative cause of death (as best as possible): | Text |
